# Cultural Adaptation of a One-Day Online Cognitive Behavioral Therapy Group Workshop for Postpartum Depression in Türkiye: A Pilot Randomized Controlled Trial

**DOI:** 10.1192/j.eurpsy.2026.12041

**Published:** 2026-07-31

**Authors:** B. Kalaça, R. J. Van Lieshout, P. Seçgin, P. Boran, H. E. Barış, M. C. Us, A. Sakallı Kani

**Affiliations:** 1 Department of Psychiatry, Marmara University School of Medicine, İstanbul, Türkiye; 2 Department of Psychiatry and Behavioural Neurosciences, McMaster University, Hamilton, Canada; 3 Department of Psychology, Istanbul Sabahattin Zaim University; 4 Department of Paediatrics, Division of Social Paediatrics, Marmara University School of Medicine, İstanbul, Türkiye

## Abstract

**Introduction:**

Postpartum depression affects one in five women worldwide and can have significant consequences for maternal and child well-being. International guidelines recommend cognitive behavioral therapy (CBT) as a first-line treatment for cases of mild to moderate severity. However, brief, accessible, group interventions for low- and middle-income countries remain limited. To address this gap, we set out to culturally adapt a one-day online CBT-based group workshop developed in North America for use in Türkiye.

**Objectives:**

To culturally adapt a one-day online CBT-based group workshop for women in Türkiye with postpartum depression, and to evaluate its feasibility, acceptability, and preliminary effectiveness in a randomized controlled pilot trial.

**Methods:**

This randomized controlled pilot trial primarily aims to evaluate the feasibility of a culturally adapted intervention developed in accordance with Bernal’s framework, and will recruit 60 women aged 18–45 years in the first postpartum year with an Edinburgh Postnatal Depression Scale (EPDS) score ≥10. Participants will be randomized 1:1 to intervention (n=30) or waitlist control (n=30) groups. The intervention is a one-day, cognitive behavioral therapy (CBT) workshop delivered online in four modules. Assessments will be conducted at baseline and 3 months later using the EPDS, Generalized Anxiety Disorder-7 (GAD-7), Frequency of Actions and Thoughts Scale (FATS), and the TÜRBAD Satisfaction Questionnaire (TÜRBAD-SQ).

**Results:**

The intervention content has been translated into Turkish by the research team and culturally adapted according to Bernal’s framework, ensuring conceptual integrity and inclusion of culturally relevant examples and visuals. A back-translation was completed and approved by the original program developer. A pilot session will also be recorded, subtitled in English, and reviewed for supervision and feedback by this individual. Participant feedback will then be collected to assess clarity, after which recruitment into the trial will begin (in October 2025).

**Conclusions:**

This study presents the first culturally adapted, one-day, online CBT-based group intervention for postpartum depression in Türkiye. The pilot trial will provide preliminary data on feasibility, acceptability, and effectiveness, informing the design of a full-scale randomized controlled trials. The cultural adaptation process is complete, and preliminary findings will be presented.

**Disclosure of Interest:**

None Declared

